# Bending of Layer-by-Layer Films Driven by an External Magnetic Field

**DOI:** 10.3390/ijms140712953

**Published:** 2013-06-24

**Authors:** Celina M. Miyazaki, Antonio Riul, David S. Dos Santos, Mariselma Ferreira, Carlos J. L. Constantino, Marcelo A. Pereira-da-Silva, Ricardo Paupitz, Douglas S. Galvão, Osvaldo N. Oliveira

**Affiliations:** 1 Center for Natural and Human Sciences, Federal University of ABC, 09210-170 Santo André, SP, Brazil; E-Mails: mmcelinh@yahoo.com.br (C.M.M.); mariselma.ferreira@ufabc.edu.br (M.F.); 2 Applied Physics Department, Gleb Wataghin Institute of Physics, State University of Campinas, UNICAMP, C.P. 6165, 13083-970 Campinas, SP, Brazil; E-Mails: riul@ifi.unicamp.br (A.R.); galvao@ifi.unicamp.br (D.S.G.); 3 São Carlos Institute of Physics, University of São Paulo, CP 369, 13560-970 São Carlos, SP, Brazil; E-Mails: chu@ifsc.usp.br (D.S.D.S.); maps@ifsc.usp.br (M.A.P.-S.); 4 Faculty of Science and Technology, São Paulo State University, UNESP, 19060-900 Presidente Prudente, SP, Brazil; E-Mail: case@fct.unesp.br; 5 Paulista University Center, UNICEP, 13563-470 São Carlos, SP, Brazil; 6 Physics Department, IGCE, São Paulo State University, UNESP, 13506-900 Rio Claro, SP, Brazil; E-Mail: paupitz@rc.unesp.br

**Keywords:** magnetic nanoparticles, natural rubber latex, carboxymethyl-chitosan, layer-by-layer assembly, molecular dynamics

## Abstract

We report on optimized architectures containing layer-by-layer (LbL) films of natural rubber latex (NRL), carboxymethyl-chitosan (CMC) and magnetite (Fe_3_O_4_) nanoparticles (MNPs) deposited on flexible substrates, which could be easily bent by an external magnetic field. The mechanical response depended on the number of deposited layers and was explained semi-quantitatively with a fully atomistic model, where the LbL film was represented as superposing layers of hexagonal graphene-like atomic arrangements deposited on a stiffer substrate. The bending with no direct current or voltage being applied to a supramolecular structure containing biocompatible and antimicrobial materials represents a proof-of-principle experiment that is promising for tissue engineering applications in biomedicine.

## 1. Introduction

The wide variety of new nanomaterials with multifunctional properties has sparked studies aimed at biological applications such as artificial muscles [1–4], drug delivery [5–8], implants [9,10] and drug therapies within the nanomedicine paradigm [11,12]. Very stringent requirements must be satisfied for such applications, especially with regard to biocompatibility and/or biodegradability, in addition to the need of suitable mechanical properties. In almost all of these cases, the materials must be assembled in a controlled fashion, which can be performed with various experimental techniques. The layer-by-layer (LbL) method [5,13–17] based on physisorption of alternating layers of positive and negatively charged species has been proven a useful tool to adjust physicochemical properties and improve biocompatibility and mechanical properties of materials for biological applications and tissue engineering [18–23]. In addition to being suitable for functionalizing surfaces with high degree of control, it also allows for bioactivity preservation of enzymes and proteins [24,25], with which applications can be developed in bioengineering, biotechnology and biosensing. Indeed, LbL films have been used to tune the mechanical strength, cell attachment and proliferation for musculoskeletal tissue engineering [13] and in scaffolds for controlled release of drugs [26]. Jiang *et al.* obtained highly ordered free-standing structures from LbL films made with conventional polyelectrolytes and gold nanoparticles deposited onto cellulose acetate [27]. Flexible LbL films have been used as thermo-mechanical sensors, in drug delivery and optical detection [28–30] and actuation in LbL films has been also obtained with carbon nanotubes assembled on Nafion membranes [31] and with polymer/metal nanocomposites [32].

As a further step in LbL film applications, we conceived new architectures exploiting the properties of three types of material, namely natural rubber latex (NRL), carboxymethyl chitosan (CMC) and magnetic nanoparticles (MNPs). NRL was chosen due to its biocompatibility and ability to induce angiogenesis [8,33,34], in addition to the mechanical properties largely explored in industrial applications, which now allow its use in biomembranes, implants and patches for drug delivery [35–38]. CMC is a water-soluble chitosan derivative with antimicrobial, anti-bacterial, analgesic and wound healing effects, largely employed as scaffolding biomedical applications [11,39,40]. MNPs have been studied for hyperthermia treatment of cancer cells [41–44] and in drug delivery systems [45,46], especially for exploiting their biocompatibility and response to magnetic stimuli. Stimuli-responsive structures have been used in various studies where surface properties of LbL films were controlled [47–52], as in synchronized cantilever movements using an external magnetic field [29], superhydrophobic, antireflection surfaces [53] and nanoporous membranes [50]. The aim here was to identify optimized supramolecular architectures deposited onto flexible membranes amenable to exhibit deformation under low external electromagnetic fields, with no need of passing an electric current through the device, which might be troublesome depending on the desired application. Furthermore, synergy can be sought upon combining two biocompatible materials in a single coating, with a material able to kill bacteria (CMC) and another that may induce angiogenesis (NRL). We developed LbL structures able to bend flexible substrates in response to an external magnetic field with a totally reversible behavior, which have great potential for biomedical applications. In addition, we wished to address the challenge of providing—even if with only a semi-quantitative treatment—an explanation for the movement of the functionalized membranes under the magnetic field. This was achieved using molecular dynamics in a fully atomistic model, which allowed us to correlate the mechanical response with the number of deposited layers in the LbL films. Though this modeling may appear of interest from the point of view of basic physics only, we argue that the success in the design of nanodevices may increasingly depend on establishing realistic models to explain their usually complex responses.

## 2. Results and Discussion

A detailed study of the adsorption kinetics of the sequential LbL assembly was carried out to determine optimized immersion times for a full layer to be built. The stability of the magnetic nanoparticles in solution was induced by using CMC in two ways: (i) CMC was added during the MNP synthesis (MNP-CMC_1_) and (ii) through a physical mixture of MNPs and CMC (MNP-CMC_2_), obtained after 30 min in an ultrasound bath. The optimized immersion times for the LbL film fabrication were 600, 420, 300 and 300 s for NRL, CMC, MNPs and MNP-CMC_2_, respectively (results not shown), with an immersion time of 120 s for MNP-CMC_1_, since longer times led to MNP precipitation during film fabrication.

**Figure 1:**
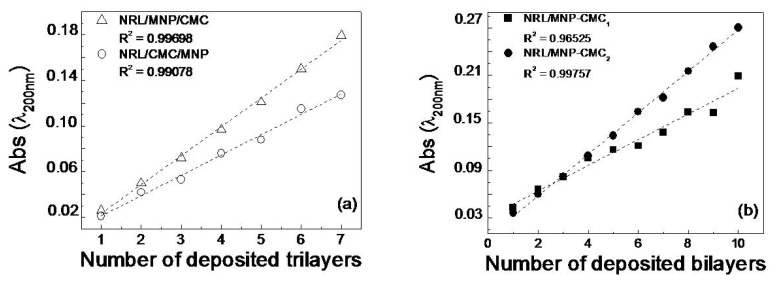
Growth of containing layer-by-layer (LbL) films: (**a**) Natural rubber latex/magnetite (Fe_3_O_4_) nanoparticles/carboxymethyl-chitosan (NRL/MNP/CMC) and NRL/CMC/MNP; (**b**) NRL/MNP-CMC_1_ and NRL/MNP-CMC_2_.

The following LbL film architectures were fabricated: NRL/MNP-CMC_1_, NRL/MNP-CMC_2_, NRL/MNP/CMC and NRL/CMC/MNP. The UV-Vis. absorption increased linearly with the number of trilayers and bilayers for all film architectures, as indicated in Figure 1. Film growth was monitored by measuring optical absorption at 200 nm where NRL absorbance is maximum. The linear behavior indicates that the same amount of material was adsorbed in each deposition step, with the highest adsorption for NRL/MNP/CMC and NRL/MNP-CMC_2_, which were further confirmed in the AFM analysis. The stability of these NRL/MNP/CMC and NRL/MNP-CMC_2_ LbL films was checked by keeping them in ultrapure water under moderate stirring for 10 min. Seven of these immersions in water were performed, with a UV-Vis absorption spectrum taken after each dipping. There was a decrease of only 0.8% in the absorbance, thus demonstrating good adhesion of the LbL films to the flexible substrates. All the three materials used are expected to be negatively charged at the pH of the experiments, and, therefore, secondary forces (e.g., hydrogen bonding, hydrophobic and van der Waals interactions [54]) should drive the thermodynamic process for spontaneous adsorption in bilayers and trilayers described here, with the components being physisorbed in supramolecular structures.

**Figure A1:**
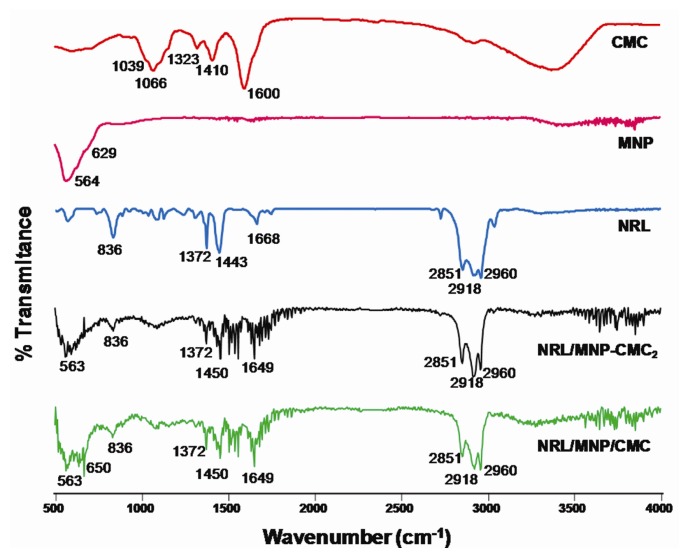
FTIR spectra for NRL/MNP/CMC and NRL/NMP-CMC_2_ LbL films and cast films of CMC, MNP and NRL. The MNP band at 563 cm^−1^ appears for both LbL films, while the band at 629 cm^−1^ for the cast film appears in the trilayer LbL film at 650 cm^−1^. This band is not evident in the LbL film because each bilayer was obtained with a mixed MNP and CMC solution, sonicated 30 min before using, and under such conditions CMC might form a structure surrounding the MNP, thus causing changes in the vibrational spectrum. In other words, the shift is probably due to steric hindrance, being typical in LbL films owing to the intimate contact between the film components [55].

Because of a more effective adsorption, we chose the NRL/MNP/CMC and NRL/MNP-CMC_2_ systems for further studies. The presence of the film components in these LbL architectures was confirmed in the FTIR spectra in Figure A1. The spectra for NRL/MNP/CMC and NRL/MNP-CMC_2_ LbL films deposited onto ZnSe substrates displayed the same bands of the neat materials, with a few differences caused by molecular-level interaction in the LbL film owing to the intimate contact of the film components [55]. For instance, the prominent band assigned to carboxylate groups of CMC is less intense in the LbL films, as these charged groups might be involved in interactions responsible for the film formation.

**Figure 2:**
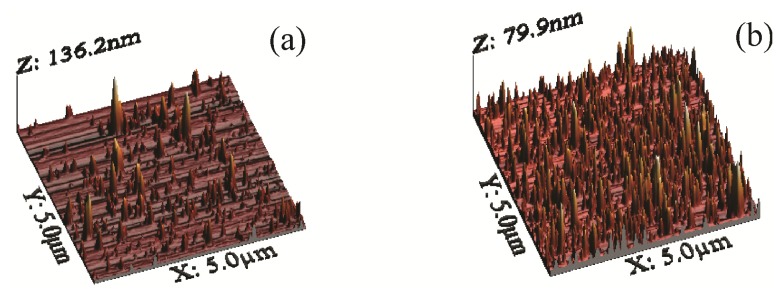
3D atomic force microscopy (AFM) height micrographs: (**a**) Five trilayers of NRL/MNP/CMC; (**b**) Five bilayers of NRL/MNP-CMC_2_.

Atomic force microscopy (AFM) images for NRL/MNP/CMC and NRL/MNP-CMC_2_ LbL films in Figure 2 display agglomerates of different sizes, distributed over the surface for both films. The MNPs appear in larger amounts and better distributed in the NRL/MNP-CMC_2_ bilayer, which is consistent with the higher adsorption inferred from Figure 1. This means that the MNPs were stabilized in the CMC solution during the LbL film formation, while in the trilayer CMC just covers the deposited MNP layer. The root mean square (RMS) roughness was 8.6 and 8.4 nm for NRL/MNP/CMC and NRL/MNP-CMC_2_, respectively, which is much higher than that of LbL films with no MNPs (RMS roughness ~0.8 nm for a neat LbL NRL film, and RMS ~0.7 nm for a 10-bilayer NRL/CMC LbL film).

Since the RMS roughness values were close for NRL/MNP/CMC and NRL/MNP-CMC_2_, the bearing tool was used in the AFM analysis, providing the ratio between the MNPs occupied area and the total LbL area against the average height of the MNPs at the LbL film surface. Table 1 indicates that the NRL/MNP/CMC film had lower area and occupied volume, with aggregates possessing higher average height and size, being, therefore, distributed less uniformly over the LbL film surface. In contrast, the values for the NRL/MNP-CMC_2_ bilayer confirmed the smaller, better distributed agglomerates in the LbL structure. Therefore, we chose NRL/MNP-CMC_2_ to be deposited onto flexible membranes for the remaining analysis.

**Figure 3:**
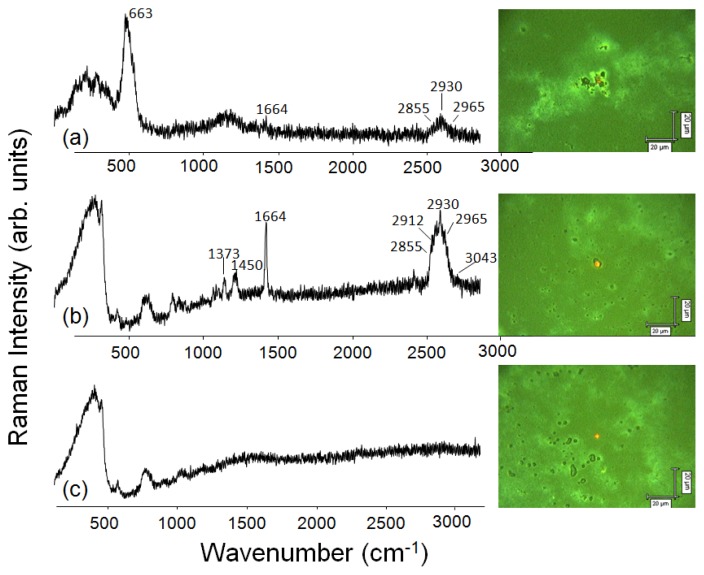
Raman spectra and optical micrographs (500× magnification) made at distinct regions of a 10-bilayer NRL/MNP-CMC_2_ LbL film. Excitation laser line: 633 nm.

Figure 3 shows the Raman spectra at three distinct regions in the NRL/MNP-CMC_2_ LbL films, together with optical micrographs acquired with 500× magnification. The 633 nm excitation laser line and the 50× objective lens lead to a spatial resolution of *ca.*1 μm^2^. The red dot indicates the place where the data were acquired. In the first spectrum (at the top), the presence of NRL and MNPs was confirmed in the LbL bilayer film, with characteristic bands at 663 cm^−1^ (Fe–O vibrations) in black aggregates for MNPs [56], and at 1664 cm^−1^ (C=C stretching of isoprene vibrations) for NRL at the lighter sites [57]. In the other region, NRL bands were predominant at *ca*. 1000 cm^−1^ assigned to C–C stretching, at 1373 and 1450 cm^−1^ due to C–H bending of CH_3_ and CH_2_, respectively, besides at 1664 cm^−1^. A zoom at the higher wavenumbers region allows one to identify bands at 2855 and 2912 cm^−1^ assigned to CH_2_ and CH_3_ symmetric stretching, respectively, and at 2930 and 2965 cm^−1^ attributed to CH_2_ and CH_3_ antisymmetric stretching, respectively. The weak band at 3043 cm^−1^ is assigned to =C–H stretching. CMC is not seen because its Raman cross section is very low for exciting lasers in the visible range, leading to undetectable signals.

**Figure 4:**
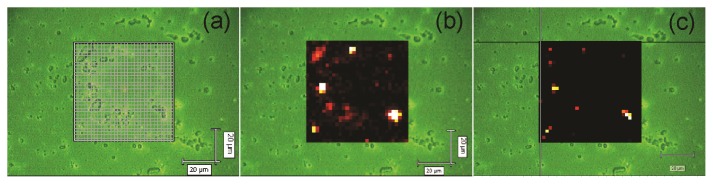
Raman mapping for a 10-bilayer NRL/MNP-CMC_2_ LbL film grown onto a quartz plate. (**a**) Scanned area (60 μm × 60 μm, step 2 μm); (**b**) Mapping of the 1664 cm^−1^ band, characteristic of NRL; (**c**) Mapping of the 663 cm^−1^ band, characteristic of MNPs. Excitation laser line: 633 nm.

Figure 4a depicts the area of a10-bilayer NRL/MNP-CMC_2_ LbL film onto a quartz plate, which was scanned with the 633 nm laser line and 50× objective lens to obtain a Raman mapping. The latter was built by collecting Raman spectra every 2 μm (step) along an area of 60 μm × 60 μm (*ca.* 1 μm^2^ spatial resolution) and then plotting the intensity of a certain peak where brighter spots correspond to higher intensities (higher material concentration). Figure 4b shows the 1664 cm^−1^ band as bright regions in the scanned area, indicating NRL agglomerates. In Figure 4c the mapping of the 663 cm^−1^ band shows the MNP aggregates at the brighter regions. In addition, there are a few bright spots in Figure 4c, indicating only a few aggregates of MNPs in the NRL matrix. Note that NRL retains its particulate form with distinct aggregate sizes even in LbL films [58].

The spots containing higher concentrations of NRL (brighter spots in Figure 4b) also present the Raman signal from aggregated MNPs (bright spots in Figure 4c), showing that MNPs are trapped within NRL agglomerates, forming aggregates of micrometer size. One could speculate that the distribution of the MNPs in the Raman mapping are not as homogeneous as shown by AFM (Figure 2), leading to inconsistent results. However, the latter must be attributed to the different spatial resolution of these techniques (AFM and micro-Raman). In the regions with well distributed MNPs (as shown in the AFM image in Figure 2) the concentration of material is not sufficient for detection via micro-Raman scattering, thus yielding dark regions in the Raman mapping (Figure 4c), *i.e.*, the micro-Raman is able to detect only aggregates of MNPs. Therefore, the few bright spots in Figure 4c are indeed consistent with a homogeneous distribution of MNPs. For instance, there were 27 spectra corresponding to MNPs out of 900 spectra recorded for the scanned area.

### LbL Coating of Flexible Membranes

**Figure 5:**
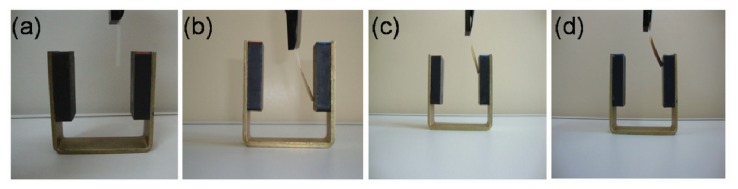
Flexible membranes bent by an external magnetic field: (**a**) Cellophane paper without LbL assembly; (**b**–**d**) Cellophane paper, transparency sheet, and NRL membrane, respectively, coated with NRL/MNP-CMC_2_ LbL film.

15-layer NRL/MNP-CMC_2_ LbL films were deposited onto cellophane paper and transparency sheets. Due to its higher density, a 32-layer NRL/MNP-CMC_2_ LbL film was deposited onto NRL membranes, all of them responding positively to an external magnetic field, as shown in Figure 5.

**Figure 6:**
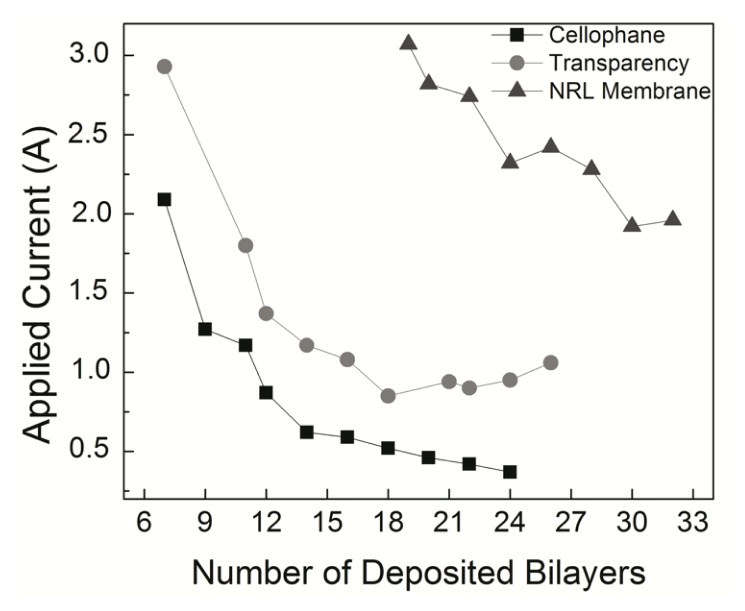
Electric current required to bend a flexible membrane placed 2 mm from a magnetized iron core. With thicker films a lower current is required.

The lowest amount of deposited material required for a response to an external magnetic field able to bend the flexible membranes positioned 2 mm from a magnetized iron core was seven bilayers of NRL/MNP-CMC_2_. This was inferred from Figure 6, where the applied current required for bending is plotted against the number of deposited bilayers. The results considering thickness, mass and measured current of the membranes onto which the LbL films were deposited, are summarized in Table 2. As expected, heavier membranes needed higher currents to be bent, and with increasing thickness of the LbL films lower currents were needed (mA in some cases) to produce the same physical effect (mechanical bending). This is due to the increased adsorption of MNPs in the LbL film, confirming the incorporation of magnetite, consequently requiring lower external magnetic fields to move the membranes. It is worth mentioning that without the incorporation of MNPs in the LbL structure, no magnetic effect (or bending) could be observed. Also, when the electric field was removed, the membrane recovered its former positioning immediately.

The mechanical properties of LbL films have been studied [59,60] normally aimed at practical applications, but in some cases including analysis of basic elasticity phenomena. Particularly relevant for the present work are the papers by Jiang *et al.* [61], where a reduced elasticity was reported for films thinner than the lengths of the domains responsible for the mechanical properties, and Jiang and Tsukruk which brings a review on the characteristics of freestanding films fabricated via the LbL technique [28]. With regard to modeling the elastic properties, a one-dimensional finite element representation was used for calculating the elasticity in layer-by-layer systems [62]. However, simulations at the atomistic level have not been presented in the literature, which is done here.

The experimental data in Figure 6 and Table 2 can be understood as a competition process between the magnetic force tending to bend the structure against the elastic restoration force that opposes it. The net response will depend on many factors, such as the number of layers, number of magnetic particles and force intensities. In order to test this hypothesis, we carried molecular dynamics (MD) simulations based on a fully atomistic model, where these parameters can be varied and the mechanical deformations analyzed. It should be stressed that a similar analysis could also have been done using finite elements or coarse grain models. However, the number of unknown parameters would imply in an extensive number of simulations, being difficult to isolate the main contributions to the observed behavior. With MD we have a very flexible framework to probe interactions between layers, which is useful to identify and isolate the main contributions for the effects we experimentally observed. This approach can be easily adapted for other film architectures and components, thus allowing a direct comparison with similar materials. Also, an atomistic model is interesting because it is relatively easy to include a realistic description of the interlayer interactions, which is of crucial importance in the present study.

**Figure 7:**
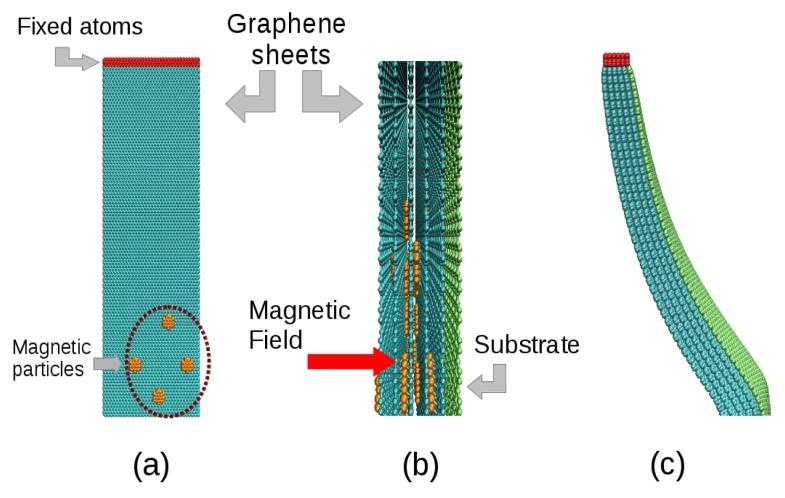
Scheme of the atomistic model used, with the case of 5-layer structures being highlighted. The membranes represented in cyan color containing magnetic nanoparticles (in orange) were constructed as hexagonal lattices. The substrate is represented by a green layer which was made stiffer than the graphene-like membranes to mimic the rigid substrates. (**a**) Front view; (**b**) Side view of the model and its internal structure; (**c**) A five-membrane system bent after application of magnetic force on the nanoparticles.

**Figure 8:**
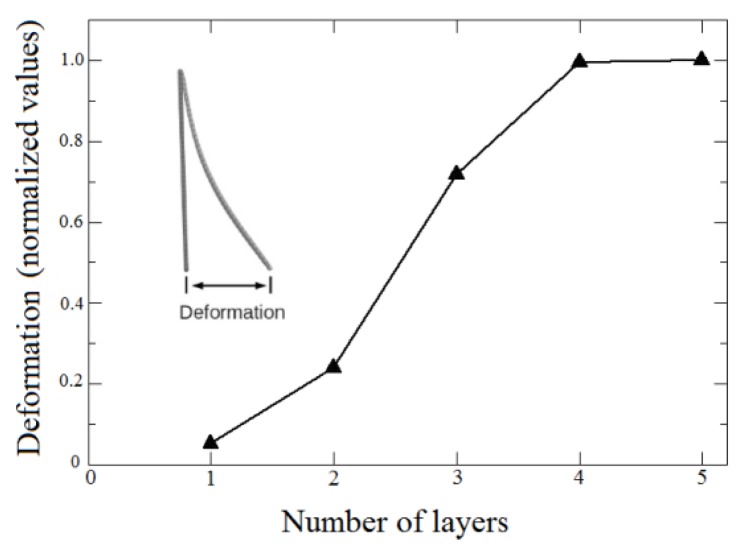
Bending deformation as a function of the number of layers. The deformation is measured as indicated in the inset.

Figure 7 depicts an overview of the atomistic model used to simulate the essential features of the experiment leading to the results in Figure 8. The model consisted of a superposition of membranes made of hexagonal graphene-like atomic arrangements. The systems considered here have sizes ranging from approximately 15,000 up to 58,000 atoms. Small atomic metallic clusters were deposited on these membranes in order to simulate the effect of magnetic nanoparticles into the system. The membranes were in close contact with a stiffer membrane, which simulates the effect of a rigid substrate. The action of a magnetic field over the nanoparticles was simulated by the application of a per-atom force on the atoms of the clusters, while the temperature was controlled by a Langevin thermostat. The use of graphene-like layers with embedded metallic clusters is believed to be a good model for the problem investigated here for the following reasons: (i) the graphene-like layers can easily be tuned (adjusting the parameters) to mimic the experimental conditions in terms of elasticity (bending/stiffness), as well as the strength of the interlayers interactions. In this way we can identify the importance of different contributions to the observed behavior. The membrane elastic behavior as well as the interlayers interactions can be varied by changing the parameters associated with bond-lengths, bond-angles and dihedrals; (ii) the nanoparticles can also be easily simulated as a cluster of metallic atoms embedded on the graphene-like layers. More importantly, the external forces can be applied only on the nanoparticles, thus realistically reproducing the experimental conditions.

The interactions between atoms in the membrane, nanoparticles and substrates were simulated using the well-known, widely tested CHARMM force field potential [63], as implemented in the Large-scale Atomic/Molecular Massively Parallel Simulator (LAMMPS) code [64]. With the model in Figure 7, it was possible to study bending as a direct function of the number of layers, as in the experiments. We considered five cases, from one up to five layers deposited on the substrates. Our results show indeed a threshold for the magnetic field to obtain the bending effect. Above this threshold, bending is proportional to the number of layers, in good qualitative agreement with the experimental data, in particular those presented in Figure 6, thus validating our working hypothesis. Also, the effect of bending on the systems is similar for four and five layers, as can be seen in Figure 8, indicative of a saturation regime. These results can be better visualized in the video in the Supplementary Material, which shows the evolution of bending as a function of the number of magnetic layers.

Finally, the experiments performed here do not allow us to determine whether stresses generated by migration of magnetic particles in the LbL films would affect the bending. In case this migration occurs, the reorganization of materials on a molecular scale could actually maximize the transduction efficiency, thus enabling hundreds of bending cycles under small magnetic external stimuli. This would be promising for sensing and actuation, especially as the reversibility of the bending process is dictated by the magnetic effect of the MNPs in the LbL films, instead of swelling, electrostatic, steric or solvation forces of the polymer layers.

## 3. Experimental Section

### 3.1. Materials

**Figure A2:**
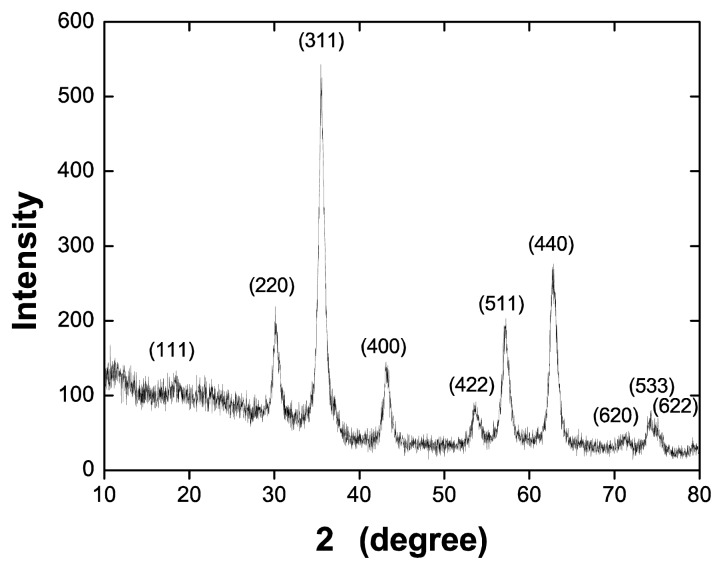
XRD diffraction patterns for the synthesized magnetic nanoparticles. Using the Scherrer equation, an average diameter of 12.6 nm was obtained for the synthesized nanoparticles.

All solutions were prepared using ultrapure water supplied by a Millipore Direct-Q system (18.2 MΩ.cm at 25 °C). Natural rubber latex was collected from *Hevea brasiliensis* RRIM 600 clones, kindly donated by EMBRAPA (São Carlos, Brazil), and stored in 20% NH_4_OH solutions to avoid coagulation. Under these conditions, *M*_w_ = 1.4 × 10^6^ g/mol [65]. Latex is a complex colloidal system composed of *cis*-1,4-isoprene particles coated with non-rubber compounds such as phospholipids, proteins, lipids and water [66]. The particles are negatively charged at high pHs and have sizes varying from 5 nm to 3 μm [58]. CMC was prepared according to the procedures of ref. [67], and had a substitution degree of 0.49 and negative charge due to its carboxylate groups. All polyelectrolytes had pH 8 adjusted by adding 0.01 M HCl or NaOH solutions. MNPs were synthesized using the co-precipitation method, in which NaOH was added to a 1:2 mixture of Fe^2+^/Fe^3+^ (15 mL of FeCl_2_ 5H_2_O 0.1 M and 30 mL of FeCl_3_ 6H_2_O 0.1 M) at pH 10, kept at 80 °C for 1 h. The product was filtered and dried in a glass desiccator. The powder was analyzed with X-ray diffraction (XRD) (Figure A2 in Appendix), from which an average diameter of 12.6 nm was obtained using the Scherrer equation [30,68,69]. MNP-CMC_1_ suspensions were prepared by adding 100 mL of a 1 mg/mL solution of CMC during the synthesis of MNPs. In addition, MNP-CMC_2_ suspensions were prepared by adding MNPs in a CMC solution, kept for 30 min. in an ultrasound bath.

### 3.2. Layer-by-Layer Assembly

The quartz substrates used were thoroughly washed in ethanol, acetone and immersed in a (1:1:5) solution of (NH_4_OH:H_2_O_2_:H_2_O) and heated at 80 °C for ~10 min. After cooling, the quartz plates were washed and stored in Milli-Q water. The flexible substrates used were cellophane paper, transparency sheets and NRL membranes (made by spreading 9 mL of neat NRL in a Petri dish heated at 60 °C till drying). These substrates were all cleaned in ethanol before use. It is worth mentioning that a layer of poly(allylamine hydrochloride) (PAH) was used as a cushion to improve the LbL growth on flexible substrates. The LbL films were mechanically fabricated by alternated immersions in the different materials using the dipping mechanism of a Langmuir trough (NIMA, Coventry, UK, model 602). We investigated four different architectures: trilayers of NRL/CMC/MNP and NRL/MNP/CMC and bilayers of NRL/MNP-CMC_1_ and NRL/MNP-CMC_2_.

### 3.3. Characterization of Layer-by-Layer Assembly

UV-Vis absorption spectroscopy measurements were performed in a Thermo Scientific, Mumbay, India, Genesys 6 and Varian, Santa Clara, CA, USA, Cary 50 Conc spectrometers. FTIR data were obtained in a Brucker, Coventry, UK, Vector 22 equipment, and Raman analysis was done using a micro-Raman Renishaw, in-Via model, with a 633 nm laserline. AFM micrographs were acquired in a Multimode Nanoscope 3a (VEECO-Digital Instruments, Santa Barbara, CA, USA) in the tapping mode with a silicon cantilever at 330 kHz and 10 μm/s scan at ambient temperature. The images were analyzed with the WSxM 5.0 software from Nanotec Electronics, Madrid, Spain [70]. The experimental setup used to analyze the actuation of flexible membranes onto which LbL films were deposited consisted of a DC generator (Instrutherm, São Paulo, Brazil, model FA-3005) associated with two coils (~700 turns each) and an iron core to amplify the magnetization effect. Flexible membranes coated with LbL films were observed against a graph paper placed 2 mm from the iron core and a DC voltage was applied until magnetic attraction was observed with the membrane moving toward the iron core. The corresponding current was plotted against the number of deposited layers in the LbL films.

## 4. Conclusions

We have tested various LbL film architectures using NRL, CMC and MNPs deposited onto quartz substrates. The transfer of the film components was confirmed using FTIR and Raman spectroscopes. Because LbL films with the NRL/MNP-CMC_2_ were the most uniform with more homogeneous distribution of MNPs, according to the AFM and micro-Raman analyses, they were further deposited on flexible substrates. This allowed the bending of flexible membranes, which increased with the amount of MNP embedded in the LbL assembly. These experimental data could be explained semi-quantitatively with a molecular dynamics model at the atomistic level, in which the films were represented by graphene-like structures containing embedded magnetic nanoparticles. Bending was the outcome of two major competitive forces, namely the magnetic force and the elastic restoration force. The successful modeling paves the way for assisting in the design of supramolecular structures used in biomedical applications, in particular with promising LbL structures that could be successfully applied as versatile interfaces able to be modulated by external stimuli. Moreover, with the use of biologically compatible materials from natural resources, as in the LbL films employed here, one may achieve a reversible actuation mechanism due to an interfacial stress promoted by MNPs in the LbL films. This might be an advantage for biomedicine, as there is no need of metallic electrodes, ionic transport, swelling of the materials or currents passing through the sample.

## Figures and Tables

**Table 1:** Morphological parameters for the LbL films. The average height of grains was estimated from the ratio between their volume and area. This procedure was adopted because the AFM image could not be segmented accurately into grains, and taking the grain size directly from the image could introduce errors due to convolution of the AFM tip.

	NRL/MNP/CMC	NRL/MNP-CMC_2_
RMS roughness (nm)	8.6	8.5
Maximum height (nm)	136.2	79.9
Average roughness (nm)	4.1	5.8
% of the ratio between the occupied area against the total area	10.7	29.2
Occupied area (10^6^ nm^2^)	2.7	7.2
Occupied volume (10^7^ nm^3^)	4.3	8.4
Average height (nm)	16.0	11.5

**Table 2:** Voltage and current values for a NRL/MNP-CMC_2_ LbL film deposited on different substrates.

Substrate	Mass (g)	Minimum number of deposited bilayers for attraction at 2 mm	Voltage (V)	Current (A)
Cellophane	0.01004	7	19.4	2.09
Transparency	0.01120	7	28.4	2.93
NRL	0.19775	19	28.9	3.07

## Appendix

A video film is available at http://www.ifi.unicamp.br/~galvao/Toto [71], which shows the results from the atomistic simulations.
